# Successful Management of Vinblastin Overdose with Exchange Transfusion: A Case Report

**Published:** 2015-04-20

**Authors:** M.R Bordbar, A Bazrafshan, M Karimi

**Affiliations:** **1. Hematology Research Center, Shiraz University of Medical Sciences, Shiraz, Iran.**

**Keywords:** Exchange Transfusion, Overdose, Vinblastin

## Abstract

Accidental overdose of chemotherapy drugs including vinblastin (VBL) have been reported in the literature. As VBL overdose is potentially fatal, we decided to introduce a 2-year-old girl affected by langerhans’ cell histiocytosis who was accidentally injected 10-times the prescribed dose of VBL (16 mg), and was saved with whole blood double exchange transfusion at 8 and 20 hrs after the accidental injection. The earliest manifestations were irritability and sinus tachycardia which alleviated after starting digoxin and at the end of the 2^nd^ exchange transfusion. Other reported adverse effects were myelosuppression, weakness of extremities, diminished deep tendon reflexes and ileus which resolved at the time of discharge from hospital on day 13 of admission. It is speculated that exchange transfusion is an effective modality in reducing the serious adverse effects of VBL overdose.

## Introduction

Vinblastine (VBL), which is a vinca alkaloid derived from leaf extracts of the periwinkle plant *Vinca rosea Linn, *is commonly used in the treatment of many childhood neoplasms including histiocytosis, testicular cancer, and Hodgkin disease. Several cases of VBL overdose have been reported in the literature that some were rescued by urgent supportive care (1-5). We report a 2-year-old girl with Langerhans’ cell histiocytosis, who was inadvertently given 10 times the prescribed dose of VBL. She experienced severe toxicities, but was successfully saved with supportive care and exchange transfusion.

## Case report

A 2-year-old girl with body weight of 8 kg, who presented with prolonged fever and generalized body pain was brought to our outpatient oncology clinic for further workup. Whole-body technetium bone scan revealed multiple sites of increased radiotracer uptake in the skeleton including skull, a few vertebral bodies, pelvis and both femoral bones. Biopsy of the iliac bone confirmed the diagnosis of Langerhans’ cell histiocytosis (LCH). Oral prednisolone, 40 mg/m2 daily in three divided doses as a 4-week course was started and she was prescribed to receive the first dose of VBL (6 mg/m2, 1.6 mg). However, the nurse admitted that she had inadvertently injected 10 times the prescribed dose, (16 mg instead of 1.6 mg). She was immediately admitted to the pediatric oncology ward for salvage therapy. The first symptoms observed were severe irritability and sinus tachycardia (heart rate 160-180 beats/min) 3-4 hours after VBL infusion. A Broviac triple lumen central venous catheter was inserted, and a 2-volume whole blood exchange transfusion was performed about 8 hours after the accident. The procedure was repeated the next day less than 20 hours after the accidental injection. Digoxin was administered soon after admission to relieve her tachycardia. The patient’s tachycardia and irritability diminished significantly after the second exchange transfusion. Folinic acid (4 mg IV every 3 hrs), vitamin B1 (50 mg PO daily), vitamin B6 (40 mg PO daily), vitamin B12 (500 µg IM stat dose), and vitamin C (50 mg IV daily) were started to reduce the neurotoxicity of VBL. Dexamethasone which was a part of her initial treatment protocol for LCH, and phenytoin (40 mg IV every 12 hrs) to induce cytochrome-P mediated hepatic metabolism of VBL were started the day after her admission to the hospital. On the second day of admission, she developed thrombocytopenia, with the nadir (24000/mm3) on day 4, and persisted through her admission. On day 6, she was appeared to show leucopenia, so granulocyte colony stimulating factor was injected daily which helped her to recover the bone marrow suppression. The most bothering symptoms observed were severe constipation, abdominal distension and ileus which resulted in respiratory distress and tachypnea. They occurred on the 5th day, and she was managed with polyethylene glycol, and motilium syrups and warm saline enema. Other manifestations noticed were alopecia, weakness of lower extremities and diminished deep tendon reflexes. She was discharged from the hospital on day 13 with good general health, and most of the symptoms were successfully relieved by appropriate management. She is still alive, and is completing her treatment course under regular fellow up.

## Discussion

VBL exerts its cytotoxic effect by binding to tubulin, and inhibiting mitotic spindle formation, thus causing metaphase arrest during mitosis (6). Myelosuppression is the dose-limiting toxic effect of VBL. Mucositis and gastrointestinal disturbance are also frequently observed. Other reported adverse effects in cases of VBL overdose are inappropriate ADH secretion (SIADH), cardiac toxicity, dermatitis, fever, hair loss, central and peripheral neuropathy, myalgia, seizure, coma, and paralytic ileus (2-5).

VBL has poor bioavailability and is more than 80% protein-bound. The long terminal half-life and the large steady-state volume of distribution lead to avid and extensive tissue binding of the drug. It is mainly eliminated by hepatic metabolism and biliary excretion. Drugs that induce CYP3A4, such as anticonvulsants, and corticosteroids can help more rapid drug elimination (6). That’s why we started dexamethasone, and phenytoin in our patient soon after 1st exchange transfusion. 

Overdose of the drugs in vinca alkaloid family such as VBL, vincristine (VCR) and vinorelbine have been described previously with different outcomes (1-5, 7-9). They are potentially fatal if urgent and timely appropriate managements are not provided. Unfortunately, there is no proven specific antidote for these drugs. The treatments are mainly supportive to alleviate the predominant drugs’ adverse effects such as neurotoxicity, ileus and myelosuppression. Vitamin supplementations including folinic acid, thiamin, cyanocobalamin, pyridoxine, and ascorbic acid, has been proposed to be of benefit (7). Other possible therapies such as glutamic acid, citrovorum factor, steroid have been recommended in previous experiences (2,5). An anti-vinca alkaloid antibody was introduced a few years ago, but its clinical applicability warrants further investigations (10). 

The role of plasma exchange in the treatment of various poisonings has been largely studied and is well-established. Its ability to remove toxins which are highly protein-bound and with large molecular weights is unique compared to hemodialysis. Theoretically, a substance which is highly protein bound (> 80%), and with small volume of distribution (< .02 L/kg) is efficiently removed by plasma exchange (11). It has been successfully tried previously in some cases of VCR and VBL overdoses (4, 12). 

Two-volume exchange transfusion as an alternative to therapeutic plasma exchange has been rarely used in cases of vinca alkaloid overdoses. Kosmidis reported 3 cases of acute lymphoblastic leukemia with VCR overdose that were treated with exchange transfusion. Two of them survived with dramatic decline in postexchange VCR levels (13). Our patient was also successfully salvaged with timely double exchange transfusion. Although she developed some manifestations of VBL overdose such as myelosuppression, neuropathy and ileus, they were all transitory, and could be handled with supportive treatments.

In conclusion, as there is no proven antidote for saving cases of VBL overdose, plasmaphresis or exchange transfusion can be safely used to remove this potentially fatal drug, provided that they are implemented in a timely manner.
